# Adjuvant therapy for renal cell carcinoma: lessons from past failures and new opportunities in the era of immune checkpoint inhibition

**DOI:** 10.3389/fimmu.2026.1816253

**Published:** 2026-04-27

**Authors:** Zengguang Liu, Xiaofeng Cong, Ziyi Liu, Jiaxin Yin, Chen Chen, Ziling Liu

**Affiliations:** Department of Cancer Center, The First Hospital of Jilin University, Changchun, China

**Keywords:** adjuvant therapy, biomarker-driven stratification, immune checkpoint inhibitors, pembrolizumab, precision oncology, renal cell carcinoma

## Abstract

Renal cell carcinoma (RCC) has historically posed a significant therapeutic challenge in the adjuvant setting. Although surgical resection remains the cornerstone of curative-intent treatment for localized disease, a substantial proportion of patients with high-risk pathological features will experience recurrence following nephrectomy. Over several decades, multiple adjuvant strategies, including cytokine-based immunotherapy and vascular endothelial growth factor (VEGF)-targeted agents, failed to deliver consistent disease-free or overall survival benefits, often limited by toxicity and poor tolerability. These repeated disappointments reinforced the perception that effective adjuvant therapy in RCC was elusive. The emergence of immune checkpoint inhibition has fundamentally reshaped this landscape, with adjuvant pembrolizumab demonstrating disease-free and overall survival benefit in selected patients with resected clear-cell RCC at increased risk of recurrence, including those with M1 no evidence of disease, thereby establishing a new standard of care for certain high-risk populations. In contrast, several contemporaneous trials evaluating alternative immune checkpoint strategies failed to meet primary endpoints, underscoring that benefit is not class-wide and is highly dependent on patient selection and disease biology. Against this background, rather than simply tracing the historical evolution of adjuvant therapy, this review examines the broader challenges of adjuvant management after RCC resection, including why most postoperative approaches failed, how current evidence has redefined care for selected patients, and what barriers remain to optimizing outcomes. Particular emphasis is placed on recurrence risk assessment, patient selection, treatment-related toxicity, and the need for biomarker-driven, precision-based strategies to guide the next generation of adjuvant treatment.

## 1 Introduction

Renal cell carcinoma (RCC) is the primary histologic subtype of kidney cancer, with clear cell renal cell carcinoma (ccRCC) being the most common form and the cause of the majority of cancer-related deaths (1). Epidemiological data indicate that there were approximately 435,000 new cases and 155,700 deaths from RCC worldwide in 2022 (2, 3). In terms of relative frequency, RCC accounts for approximately 2.4% of all cancer diagnoses globally (4). For patients with clinically localized disease, surgical resection by means of partial nephrectomy (nephron-sparing surgery) or radical nephrectomy remains the cornerstone of curative-intent treatment and is strongly endorsed by major clinical practice guidelines (5–7). Despite excellent short-term oncologic control in many patients, long-term outcomes following surgery alone remain suboptimal in a clinically meaningful subset. Large population-based and institutional studies have consistently shown that approximately 35% of patients with localized RCC (stage II-III) ultimately develop disease recurrence after complete surgical resection, while in patients with very-high-risk clear-cell disease, placebo-group data from adjuvant trials suggest a 5-year recurrence-free survival of approximately 50%, indicating a substantial risk of postoperative relapse (8, 9).

In the contemporary adjuvant ccRCC setting, clinically relevant high-risk populations include patients with intermediate-high risk and high-risk clear-cell RCC, as well as those with M1 no evidence of disease (M1 NED) after complete resection of synchronous or metachronous metastatic lesions (5, 7).These categories are typically defined by adverse clinicopathologic features, including locally advanced primary tumors, regional lymph node involvement, high nuclear grade, and sarcomatoid differentiation (5, 7). These patients experience a particularly poor prognosis once relapse occurs, as recurrent RCC is largely incurable and typically requires lifelong systemic therapy aimed at durable disease control and survival prolongation rather than cure (5, 7). In this context, adjuvant therapy administered in the postoperative, disease-free setting offers a strong theoretical rationale. By targeting micrometastatic disease prior to overt clinical relapse, adjuvant treatment has the potential to reduce the risk of recurrence, prolong survival, and possibly increase cure rates. Despite this compelling rationale, effective adjuvant strategies for RCC remained elusive for decades.

The development of adjuvant therapy in RCC has historically been constrained by the distinct biological characteristics of the disease. RCC is intrinsically resistant to conventional cytotoxic chemotherapy and demonstrates limited sensitivity to radiotherapy, thereby restricting the applicability of traditional adjuvant treatment paradigms that have proven effective in other solid malignancies. Consequently, systemic postoperative treatment options were historically limited to immunomodulatory or targeted therapeutic approaches. Over the past three decades, multiple phase III trials evaluating adjuvant vascular endothelial growth factor receptor (VEGFR) tyrosine kinase inhibitors (TKIs), based on the premise that RCC is a highly angiogenesis-driven malignancy, largely failed to demonstrate consistent improvements in survival outcomes and were frequently limited by treatment-related toxicity and high rates of treatment discontinuation (10, 11). More recently, perioperative immune checkpoint strategies have yielded heterogeneous results. For example, a large randomized trial evaluating a single preoperative dose of nivolumab and adjuvant treatment with nivolumab following surgery did not improve recurrence-free survival when compared to surgery only (12). In contrast, the field has entered a new phase with evidence that adjuvant pembrolizumab provides not only a disease-free survival (DFS) benefit but also a statistically significant overall survival (OS) advantage in patients with resected ccRCC at increased risk of recurrence, including those with M1 NED (13). Taken together, these findings underscore both the longstanding challenge of translating compelling biological rationale into meaningful clinical benefit in RCC and the need for refined patient selection and biomarker-informed strategies to optimize the risk-benefit balance of adjuvant therapy (7).

**Figure 1:**
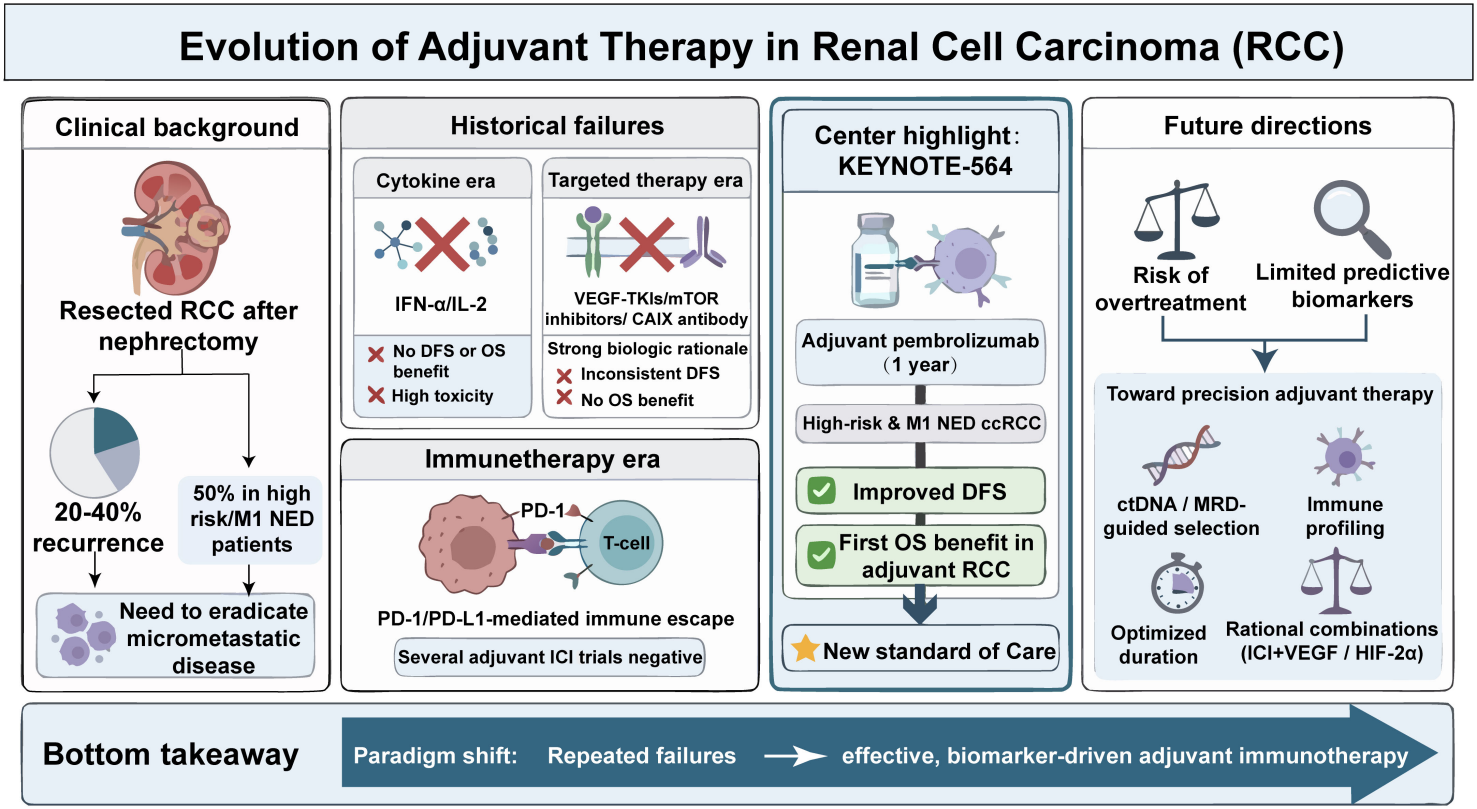
Evolution of adjuvant therapy in renal cell carcinoma (RCC). Despite curative-intent nephrectomy, a substantial proportion of patients with localized RCC experience disease recurrence, particularly those with high-risk or M1 NED features. Early adjuvant strategies, including cytokine-based and angiogenesis-targeted therapies, failed to provide consistent survival benefit. In contrast, the KEYNOTE-564 trial established adjuvant pembrolizumab as the first therapy to improve both disease-free and overall survival, marking a paradigm shift toward precision, biomarker-informed adjuvant immunotherapy. RCC, renal cell carcinoma; ccRCC, clear cell renal cell carcinoma; NED, no evidence of disease; DFS, disease-free survival; OS, overall survival; IFN-α, Interferon-α; IL-2, Interleukin-2; PD-1, programmed cell death protein 1; PD-L1, programmed death ligand 1; ICI, immune checkpoint inhibitor; ctDNA, circulating tumor DNA; MRD, minimal residual disease; VEGF, vascular endothelial growth factor; HIF-2α, hypoxia-inducible factor 2α.

This review aims to do more than summarize the historical evolution of adjuvant therapy in RCC. Rather, it integrates evidence across the cytokine, VEGF-targeted, and immune checkpoint inhibitor eras to explain why most postoperative strategies failed, why pembrolizumab emerged as the first successful adjuvant option, and how these lessons may inform the next generation of perioperative treatment (as shown in **Figure 1**). We summarize the biological rationale, key clinical trials, and pivotal evidence that have shaped contemporary practice, with particular emphasis on the transition from angiogenesis-targeted approaches to immunotherapy-based paradigms. In addition, we address ongoing controversies, including optimal patient selection, risk stratification, treatment duration, and the balance between long-term benefit and treatment-related toxicity. By integrating clinical evidence with emerging biological insights, we propose a conceptual framework in which the future of adjuvant therapy for RCC shifts from empirically treating all high-risk patients toward biologically informed, biomarker-guided precision strategies.

## 2 The pre-immunotherapy era: early attempts and repeated failures

### 2.1 Cytokine-based adjuvant therapy

Before the advent of molecularly targeted agents, cytokine-based immunotherapy constituted the mainstay of systemic treatment for RCC. Interferon-α (IFN-α) and high-dose interleukin-2 (IL-2) demonstrated modest antitumor activity in metastatic RCC, with objective responses observed in a minority of patients and durable complete responses achieved in a small but clinically meaningful subset, particularly with IL-2 (14, 15). These durable remissions provided early evidence that RCC is an immunologically responsive malignancy and helped establish the conceptual foundation for investigating immunotherapy in earlier disease settings (2).

On this basis, multiple early clinical trials investigated adjuvant cytokine therapy following nephrectomy in patients with high-risk localized RCC (16–22) (as shown in **Table 1**). These studies evaluated postoperative administration of IFN-α or IL-2 with the aim of eradicating residual micrometastatic disease. However, most trials were limited by small sample sizes and substantial heterogeneity in patient selection, dosing regimens, and clinical endpoints, and were conducted prior to the adoption of contemporary risk stratification systems (7). Importantly, none of these studies demonstrated a significant improvement in DFS or OS (16, 17, 21). Beyond the absence of efficacy, cytokine-based therapy was associated with considerable toxicity, which substantially limited its tolerability in the adjuvant setting. Frequently reported adverse events included flu-like symptoms, severe fatigue, hypotension, and neuropsychiatric disturbances, whereas high-dose IL-2 carried a risk of severe capillary leak syndrome, multiorgan dysfunction, and treatment-related mortality (16, 23, 24). Collectively, randomized trials conducted during the cytokine era failed to demonstrate a reproducible DFS or OS benefit for adjuvant IFN-α, IL-2, or their combinations in RCC, and treatment-related toxicity and poor tolerability further constrained their clinical applicability.

**Table 1:** Major clinical trials of cytokine-based adjuvant therapy in renal cell carcinoma.

Study / year	Phase	Population	Enrolled patients	Treatment regimen	Control group	Primary endpoint(s)	Key efficacy outcome	Toxicity summary	
Pizzocaro et al., 2001 (17)	III	Robson stage II–III RCC after radical nephrectomy	247	Recombinant IFN-α2b (adjuvant)	Observation	OS, EFS	No significant OS and EFS benefit	Flu-like symptoms, fatigue; treatment discontinuation common	
Messing et al., 2003 (16) (ECOG/Intergroup)	III	High-risk resected RCC	283	IFN-α-NL	Observation	DFS, OS	No improvement in DFS or OS	Grade 3–4 toxicities including fatigue, myelosuppression, neuropsychiatric effects	
Clark et al., 2003 (CWG) (19)	II/III	High-risk RCC after nephrectomy	69	High-dose bolus IL-2	Observation	DFS, OS	No survival benefit	Severe systemic toxicity consistent with HD IL-2; inpatient administration required	
Atzpodien et al., 2005 (20) (DGCIN)	III	High-risk localized RCC	203	sc IL-2 + sc IFN-α2a + iv 5-FU	Observation	DFS, OS	No significant DFS or OS benefit	Dose reductions required to limit toxicity; fatigue, hypotension, flu-like symptoms	
Hinotsu et al., 2013 (18)	III	Stage II–III RCC after nephrectomy	107	Natural IFN-α (1 year)	Observation	PFS	No PFS benefit	Moderate toxicity; prolonged treatment burden	
Passalacqua et al., 2014 (22) (POLAR-01)	III	Resected RCC at increased risk	310	Low-dose IL-2 + IFN-α	Observation	RFS, OS	No improvement in RFS or OS		Better tolerated than HD regimens but limited efficacy
EORTC 30955 / NCRI (Aitchison et al., 2014) (21)	III	High-risk RCC after surgery	309	IL-2 + IFN-α + 5-FU	Observation	DFS, OS, QoL	No DFS or OS benefit		Significant cumulative toxicity; QoL negatively affected

RCC, renal cell carcinoma; IFN-α, interferon-alpha; IL-2, interleukin-2; DFS, disease-free survival; RFS, recurrence-free survival; OS, overall survival; PFS, progression-free survival; QoL, quality of life; sc, subcutaneous; iv, intravenous.

Consequently, cytokine-based adjuvant therapy was ultimately abandoned and was not incorporated into routine clinical practice. These negative experiences underscored the inherent challenges of translating immunologic activity observed in advanced disease into effective adjuvant strategies and highlighted the need for more precise, mechanism-driven approaches, which would later be realized with the advent of immune checkpoint inhibition.

Beyond the lack of survival benefit, the failure of cytokine-based adjuvant therapy also highlighted a broader conceptual challenge in RCC. Early trials largely assumed that immunologic activity observed in metastatic disease could be directly translated to the minimal residual disease setting. However, these studies were conducted before modern risk stratification and lacked biological markers capable of identifying patients with true micrometastatic disease. These limitations illustrate an early lesson in RCC adjuvant therapy: therapeutic efficacy observed in advanced disease does not necessarily translate into postoperative benefit, particularly when some patients may already be adequately treated by surgery alone and appropriate biological selection is lacking.

## 3 The rise and disappointment of targeted therapy

### 3.1 Biological rationale for VEGF-targeted adjuvant therapy

Inactivation of the von Hippel–Lindau (VHL) tumor suppressor gene represents a defining molecular event in clear cell renal cell carcinoma (ccRCC) and was instrumental in establishing angiogenesis as a central hallmark of this disease (25). Loss of functional pVHL disrupts cellular oxygen-sensing homeostasis, leading to constitutive stabilization of hypoxia-inducible factors (HIFs) and subsequent transcriptional upregulation of pro-angiogenic mediators, most notably vascular endothelial growth factor (VEGF), thereby promoting endothelial proliferation, pathological neovascularization, and tumor progression (26–28). In addition to its central role in angiogenesis, VEGF signaling contributes to an immunosuppressive tumor microenvironment by impairing dendritic cell maturation, reducing T-cell infiltration, and promoting regulatory immune populations (29); therefore, VEGF inhibition may enhance antitumor immunity and provide a mechanistic basis for combination strategies with immune checkpoint inhibitors.

These mechanistic insights directly informed therapeutic development. Multi-targeted VEGF receptor tyrosine kinase inhibitors (VEGFR-TKIs) rapidly emerged as a cornerstone of systemic treatment for metastatic RCC, as randomized phase III trials demonstrated clinically meaningful improvements in PFS and objective response rates compared with cytokine-based therapy or placebo. In the first-line setting, sunitinib significantly prolonged PFS compared with IFN-α (11 months *vs.* 5 months; hazard ratio [HR], 0.42; 95% confidence interval [CI], 0.32-0.54; *P* < 0.001) (30). In previously treated advanced ccRCC, sorafenib improved PFS relative to placebo (5.5 months *vs.* 2.8 months; HR = 0.44; 95% CI, 0.35-0.55; *P* < 0.01) (31). Similarly, pazopanib significantly extended PFS compared with placebo in patients with advanced or metastatic RCC (9.2 months *vs.* 4.2 months; HR = 0.46; 95% CI, 0.34-0.62; *P* < 0.0001) (32). Collectively, these studies established blockade of the VEGF signaling pathway as a foundational therapeutic principle in RCC (6, 7).

Given these advances in advanced disease, a strong biological and clinical rationale emerged for evaluating VEGF-targeted agents in the adjuvant setting. The underlying hypothesis was that postoperative anti-angiogenic therapy could suppress the outgrowth of occult micrometastatic disease following nephrectomy, which is thought to be angiogenesis-dependent during early colonization, thereby reducing the risk of recurrence and improving long-term clinical outcomes.

### 3.2 Key trials of adjuvant VEGF-targeted therapy and mTOR inhibition

Building on these theoretical foundations and assumptions, multiple large, randomized phase III trials were conducted to evaluate angiogenesis inhibitors targeting the VEGF pathway and, subsequently mTOR inhibitors, as postoperative (adjuvant) therapy in patients with resected RCC who remained at substantial risk of relapse (9–11, 33–36) (as shown in **Table 2**). Collectively, these studies were driven by the robust clinical activity of VEGF-targeted TKIs and mTOR inhibitors in metastatic disease, yet they underscored the challenges of translating efficacy into the disease-free setting, where treatment tolerability, treatment discontinuation, and maintenance of dose intensity critically influence clinical outcomes.

**Table 2:** Major clinical trials of targeted therapy in adjuvant RCC.

Trial	Phase	Population	Regimen	Primary endpoint	Main results	Toxicity
ASSURE (ECOG-ACRIN E2805) (10)	III	Resected high-risk, non-metastatic RCC	Sunitinib *vs.* Sorafenib *vs*. Placebo	DFS	No DFS or OS benefit for sunitinib or sorafenib *vs.* placebo; high rates of dose modification/discontinuation limited dose intensity.	Substantial TKI-class AEs; frequent dose reductions/interruptions and treatment discontinuation
S-TRAC (33, 35)	III	Resected high-risk clear-cell RCC	Sunitinib *vs.* Placebo	DFS	DFS improved with sunitinib *vs.* placebo; OS not improved in available analyses; clinical uptake limited by toxicity and lack of OS signal.	High burden of grade ≥3 AEs and discontinuations; typical sunitinib toxicities (hypertension, fatigue, diarrhea, hand–foot syndrome, cytopenias)
PROTECT (11)	III	Resected localized/locally advanced RCC at increased recurrence risk	Pazopanib *vs.* Placebo	DFS	Did not meet primary DFS endpoint (interpretation influenced by dose reductions and lower delivered dose intensity); final analyses did not establish OS benefit.	Prominent tolerability issues leading to dose reductions/interruptions; common pazopanib AEs include hepatotoxicity, hypertension, diarrhea, fatigue.
ATLAS (34)	III	Resected RCC at risk of recurrence	Axitinib *vs.* Placebo	DFS	Stopped early for futility; no significant DFS benefit in overall population (exploratory signals in highest-risk subsets were not practice-changing).	TKI-class AEs; hypertension, diarrhea, fatigue, hand–foot syndrome; dose modifications and discontinuations occurred.
SORCE (36)	III	Resected intermediate-/high-risk RCC	Sorafenib (1 year) *vs.* Sorafenib (3 years) *vs.* Placebo	DFS	No DFS/OS improvement with sorafenib (either duration) *vs.* placebo; prolonged therapy limited by tolerability/dose intensity.	Significant chronic TKI toxicity over long exposure; hand–foot syndrome, diarrhea, fatigue, hypertension; notable dose reductions/interruptions.
EVEREST (SWOG S0931) (9)	III	Resected intermediate-high / very-high-risk RCC (broader histology allowed)	Everolimus *vs.* Placebo	RFS/DFS (protocol-defined)	Overall, not practice-changing: no clear improvement in the full eligible population by prespecified statistical criteria; subset signals reported in very-high-risk groups; no clear OS impact.	mTOR-inhibitor AEs with meaningful grade ≥3 toxicity; stomatitis/mucositis, infections, rash, hyperglycemia/hyperlipidemia, pneumonitis; discontinuations occurred.
RESORT (38)	II (randomized)	M1 NED after complete metastasectomy	Sorafenib *vs.* Observation	RFS/DFS	No RFS benefit *vs*. observation; did not support adjuvant sorafenib in M1 NED.	Similar sorafenib toxicity profile; chronic AEs and discontinuations reduce feasibility in disease-free setting.
ECOG-ACRIN E2810 (39)	III	M1 NED after complete metastasectomy	Pazopanib *vs.* Placebo	DFS	Negative: pazopanib did not improve DFS *vs.* placebo; reinforced that VEGF-TKIs are not effective adjuvantly in M1 NED.	Pazopanib tolerability issues; dose reductions/interruptions common; hepatotoxicity, hypertension, diarrhea, fatigue; QoL considerations important.
ARISER (40)	III	Resected high-risk clear-cell RCC	Girentuximab (anti-CAIX) *vs.* Placebo	DFS	No DFS/OS benefit; did not establish antibody-based CAIX targeting as an adjuvant approach.	Generally manageable but infusion-related/immune-type AEs can occur; overall risk–benefit unfavorable given lack of efficacy.

RCC, renal cell carcinoma; RFS, recurrence-free survival; DFS, disease-free survival; OS, overall survival; TKI, tyrosine kinase inhibitor; AE, adverse event(s); mTOR, mechanistic target of rapamycin (also known as mammalian target of rapamycin); NED, no evidence of disease; QoL, quality of life; CAIX, carbonic anhydrase IX.

In the ASSURE (ECOG-ACRIN E2805) trial, patients were randomized to receive sunitinib, sorafenib, or placebo following nephrectomy; neither active treatment arm improved DFS or OS, and treatment was frequently limited by toxicity and the need for dose modifications (10). In contrast, the S-TRAC trial (33), which enrolled a more selected high-risk population with ccRCC, reported a statistically significant improvement in DFS with adjuvant sunitinib compared with placebo (6.8 years *vs.* 5.6 years; HR = 0.76; 95% CI, 0.59-0.98; *P* = 0.03). However, no OS benefit was observed, and clinically meaningful toxicity remained a major limitation (33). Similarly, the PROTECT study evaluating adjuvant pazopanib did not meet its primary DFS endpoint with frequent dose reductions and tolerability constraints; subsequent analyses have not established a clear OS advantage for pazopanib in this setting (11, 37). The ATLAS trial assessing axitinib was terminated early for futility at a prespecified interim analysis and failed to show a significant DFS benefit in the overall study population (HR = 0.87, 95% CI, 0.660-1.147; *P* = 0.321), although exploratory analyses suggested potential benefit in the highest-risk subgroup (34). More recently, the EVEREST trial, the only phase III adjuvant study testing an mTOR inhibitor, demonstrated that postoperative everolimus did not significantly improve 5-year recurrence-free survival in the overall high-risk cohort (67% *vs.* 63%, HR = 0.85, 95% CI, 0.72-1.00; *P* = 0.051) (9). Although a benefit was observed in very-high-risk subsets, the overall result was not practice-changing, and treatment-related toxicity was substantial, with grade 3 or higher adverse events occurring in 46% of patients receiving everolimus compared with 11% in the placebo group (9).

Molecularly targeted agents have also been investigated in a distinct postoperative, disease-free population, namely patients with metastatic RCC who achieve no evidence of disease (M1 NED) after complete resection of metastatic lesions (as shown in **Table 2**). Given the particularly high risk of recurrence in this population, there was a strong rationale to assess whether VEGF-targeted therapy could eradicate residual micrometastatic disease and prolong DFS. However, available trials have consistently yielded negative results (38). In the randomized, open-label phase II RESORT study, adjuvant sorafenib did not improve recurrence-free survival (RFS) compared with observation following complete metastasectomy (*P* = 0.404) (38). Likewise, the randomized, double-blind phase III ECOG-ACRIN E2810 trial showed that adjuvant pazopanib failed to improve 3-year DFS relative to placebo in M1 NED patients (27.4% *vs.* 21.9%; HR = 0.90, 95% CI, 0.60-1.34; *P* = 0.29), with treatment burden and quality-of-life considerations further underscoring the limited clinical value of VEGF-TKIs in this disease-free setting (39). Taken together, these data indicate that VEGF-targeted TKIs have not demonstrated clinical benefit in the M1 NED adjuvant context and therefore are not supported as routine therapy following complete resection of metastatic disease.

In addition to small-molecule inhibitors, antibody-based targeted strategies have also been explored in the adjuvant treatment of RCC. A notable example is girentuximab (40), a monoclonal antibody directed against carbonic anhydrase IX (CAIX), a transmembrane protein that is highly and selectively expressed in ccRCC while exhibiting minimal expression in normal renal and non-renal tissues. This biological specificity provided a strong rationale for evaluating CAIX-targeted therapy in the postoperative, disease-free setting.

The ARISER trial was a randomized, double-blind, placebo-controlled study that enrolled patients with high-risk ccRCC following nephrectomy to receive adjuvant girentuximab or placebo (40). Despite a compelling biological premise, the trial failed to demonstrate an improvement in DFS (HR = 0.97, 95% CI, 0.79-1.18) or OS (HR = 0.99, 95% CI, 0.74-1.32) compared with placebo. These negative findings indicated that CAIX-targeted antibody therapy did not translate into meaningful clinical benefit in the adjuvant setting, and girentuximab was therefore not incorporated into routine postoperative management of RCC.

Taken together, these negative or equivocal results underscored the limitations of extrapolating efficacy from metastatic disease to the micrometastatic postoperative context and highlighted that biologic activity alone is insufficient in the absence of acceptable tolerability and durable clinical benefit. The targeted-therapy era therefore provided critical lessons regarding patient selection, endpoint interpretation, and treatment feasibility in the disease-free setting, reinforcing the need for more effective and biologically aligned adjuvant approaches. This unmet need ultimately catalyzed the transition toward immune checkpoint inhibitor-based strategies and biomarker-informed, risk-adapted perioperative treatment paradigms. Importantly, the largely negative results of VEGF-targeted adjuvant trials likely reflect several biological and clinical factors. First, anti-angiogenic therapy primarily exerts cytostatic rather than cytotoxic effects, which may be insufficient to eradicate dormant micrometastatic disease in the postoperative setting. Second, the prolonged treatment duration required for VEGF inhibition resulted in substantial toxicity and frequent dose reductions, limiting effective drug exposure. Third, the absence of molecularly informed patient selection meant that treatment was administered to heterogeneous populations with widely variable recurrence risks. Collectively, these findings emphasize that successful adjuvant therapy requires not only active systemic agents but also appropriate biological context and patient stratification.

## 4 The immunotherapy era: KEYNOTE-564 as a milestone

### 4.1 Biological basis for immune checkpoint inhibition in RCC

RCC has long been recognized as an immunogenic malignancy, characterized by prominent immune-cell infiltration and clinical responsiveness to immune modulation (41). A central mechanism of immune escape in RCC involves the programmed cell death protein 1 and programmed death ligand 1 (PD-1/PD-L1) axis, whereby engagement of PD-L1 with PD-1 on activated T cells attenuates antitumor effector function and promotes T-cell exhaustion within the tumor microenvironment (42). Consistent with this biological framework, immune checkpoint inhibitors (ICIs) targeting PD-1 or PD-L1, either as monotherapy or in combination with other agents, have produced durable clinical responses and improved survival outcomes in metastatic RCC, thereby establishing immune checkpoint blockade as a cornerstone of contemporary systemic therapy (43, 44).

The success of immune checkpoint inhibition in advanced disease provided a strong rationale for evaluating these agents as postoperative adjuvant therapy in patients with resected RCC at increased risk of recurrence, including those with high-risk disease and those with M1 no evidence of disease after complete resection of metastatic lesions (13, 41). Beyond its recognized immunogenicity, RCC also exhibits biological features that make it conceptually well suited for immune-based intervention in the adjuvant context. Surgical resection may induce a period of immune remodeling characterized by tumor antigen release, changes in inflammatory signaling, and reorganization of systemic antitumor immunity, thereby creating a postoperative window in which residual micrometastatic disease may be more vulnerable to immune-mediated elimination than established macroscopic tumors (41). This concept is particularly relevant to the rationale for adjuvant and peri-operative immune checkpoint inhibition, as micrometastatic deposits are thought to carry lower tumor burden and a less immunosuppressive microenvironment, which may facilitate T-cell priming, expansion, and durable immune surveillance. In this setting, checkpoint blockade may be more effective at preventing or delaying recurrence by enhancing immune control over occult residual disease before overt relapse becomes clinically apparent (45, 46). In addition, the induction of durable immune memory and sustained immune surveillance by checkpoint inhibition may be particularly relevant in the adjuvant setting, where long-term immune control could prevent or delay the outgrowth of occult residual disease (41, 47). Collectively, these considerations provide a coherent biological framework supporting the clinical development of ICIs as adjuvant therapy for RCC.

### 4.2 Key adjuvant immunotherapy trials

Over the past decade, multiple randomized phase III trials have evaluated ICIs as adjuvant or perioperative therapy for RCC, reflecting a concerted effort to translate the success of immunotherapy in metastatic disease into the postoperative, disease-free setting (12, 13, 48–51) (as shown in **Table 3**). Collectively, these studies have yielded heterogeneous results, underscoring the complexity of immune modulation in early-stage RCC and the critical importance of patient selection and trial design.

**Table 3:** Major clinical trials of immune checkpoint inhibitor therapy in adjuvant RCC.

Trial	Phase	Population	Regimen	Primary endpoint	Main results	Toxicity (grade ≥3 AE / discontinuation / most common ≥3 AE)
KEYNOTE-564 (13, 48)	III	Post-nephrectomy ccRCC, increased recurrence risk, including M1 NED	Pembrolizumab *vs.* PBO for ~1 year (17 q3w cycles)	DFS	DFS benefit established; with median follow-up 57.2 months, OS improved (4-year OS rate 91.2% *vs.* 86.0%, HR 0.62).	Grade 3–4 TRAE: 18.6% *vs.* 1.2%; Discontinuation: 21.1% *vs.* 2.2%; Most common ≥3 AE: hypertension, increased alanine aminotransferase.
IMmotion010 (49)	III	Resected RCC at increased recurrence risk (clear-cell or sarcomatoid component) including M1 NED	Atezolizumab *vs.* PBO	DFS	No DFS improvement: median DFS 57.2 *vs.* 49.5 months, HR 0.93, *P* = 0.50; median follow-up 44.7 months.	Grade 3–4 AE: 27% *vs.* 21%; Discontinuation:12% *vs.*3%; Most common ≥3 AE: hypertension (2% *vs.* 4%), hyperglycaemia (3% *vs.* 2%), diarrhea (1% *vs.* 2%).
CheckMate 914 Part A (50)	III	Localized RCC at high risk after nephrectomy	Nivolumab + ipilimumab *vs.* PBO	DFS	DFS not met: median DFS not reached *vs.* 50.7 months; HR 0.92, P = 0.53; median follow-up 37.0 months.	Grade 3–5 AE: 38% *vs.* 10%; Discontinuation: 32% *vs.* 2%; (TRAE discontinuation 29% *vs.* 1%); Most common ≥3 AE: diarrhea (4% *vs.<*1%), hypophysitis (3% *vs.* 0), hepatitis (3% *vs.* < 1%).
CheckMate 914 Part B (51)	III	Localized RCC at high risk after nephrectomy	Nivolumab *vs.* PBO	DFS	DFS not met: median DFS not reached in either arm; HR 0.87, P = 0.40; median follow-up 27.0 months.	Grade 3–4 AE: 17.2% *vs.* 15%; Discontinuation: 9.6% *vs.* 1.0%; Most common ≥3 AE: hepatitis (1.2% *vs.* 1.4%), rash (0.7% *vs.* 0), adrenal insufficiency (0.7% *vs.* 0).
PROSPER ECOG-ACRIN EA8143 (12)	III	High-risk resectable RCC (clear-cell and non-clear-cell allowed); perioperative strategy	1 dose nivolumab pre-op + adjuvant nivolumab *vs.* surgery/observation	RFS	No benefit: HR 0.94, one-sided *P* = 0.32; median follow-up 30.4 *vs.* 30.1 months; stopped for futility at interim.	Grade 3–5 AE: 48% *vs.* 24%; Discontinuation: 16% *vs.* N/A; Most common ≥3 AE: anemia (6% *vs.* 5%), hypertension (7% *vs.* 2%), elevated lipase (5% *vs.* 2%).

RCC, renal cell carcinoma; AE, adverse event; ccRCC, clear cell renal cell carcinoma; PBO, placebo; TRAE, treatment related adverse event; HR, hazard ratio; RFS, recurrence-free survival.

Among these trials, KEYNOTE-564 represents a pivotal milestone (13, 48). KEYNOTE-564 was a randomized, double-blind, placebo-controlled phase III trial that evaluated pembrolizumab administered for one year following nephrectomy in patients with ccRCC at intermediate-high risk, high risk, or with M1 NED status (13, 48). The primary endpoint was DFS, with OS as a key secondary endpoint. Importantly, the trial included patients with resected metastatic disease, a population with particularly high recurrence risk. Results showed that adjuvant pembrolizumab significantly improved 2-year DFS compared with placebo (77.3% *vs.* 68.1%; HR = 0.68; 95% CI, 0.53-0.87; *P* = 0.002), providing the first robust evidence of efficacy for ICI-based adjuvant therapy in RCC (13, 48). With extended follow-up, KEYNOTE-564 further demonstrated a statistically significant OS benefit (HR = 0.62; 95% CI, 0.44-0.87; *P* = 0.005), thereby becoming the first adjuvant RCC trial to confirm an OS advantage and redefining the therapeutic standard for selected high-risk patients (13). Besides, the inclusion of patients with M1 NED and the benefit observed in this subgroup further support the potential of immune checkpoint inhibition to eradicate micrometastatic residual disease following metastasectomy (13). Moreover, accumulating evidence suggests that adjuvant pembrolizumab offers a favorable risk-benefit ratio for RCC patients at high risk of recurrence following nephrectomy (52). Additionally, emerging large-scale real-world studies have corroborated the efficacy and manageable safety of adjuvant pembrolizumab in patients with intermediate-high-risk and high-risk ccRCC (53). Although randomized trials remain the primary evidence base, emerging real-world studies such as the ARON-1 (53) analysis provide early insights into the effectiveness and safety of adjuvant immunotherapy in routine clinical practice.

In contrast, several contemporaneous phase III studies evaluating alternative ICI strategies failed to meet their primary endpoints (12, 49–51). The IMmotion010 trial, which assessed adjuvant atezolizumab in patients with resected high-risk RCC, including tumors with clear-cell or sarcomatoid features, did not demonstrate a DFS benefit over placebo (57.2 months *vs.* 49.5 months; HR = 0.93; 95% CI, 0.75-1.15; *P* = 0.5), arguing against the routine use of PD-L1 blockade in this setting (49). Similarly, CheckMate 914 Part A, which evaluated adjuvant nivolumab plus ipilimumab following nephrectomy for localized high-risk RCC, failed to improve DFS compared with placebo (not reached *vs.* 50.7 months; HR = 0.92; 95% CI, 0.71-1.19; *P* = 0.53) (50). The companion CheckMate 914 Part B trial, assessing nivolumab monotherapy, likewise did not meet its DFS primary endpoint (HR = 0.87; 95% CI, 0.62-1.21; *P* = 0.40), with results consistent with the known safety profile of nivolumab (51). Beyond purely adjuvant approaches, the PROSPER ECOG-ACRIN EA8143 trial explored a perioperative immunotherapy strategy that incorporated a single preoperative dose of nivolumab followed by postoperative administration (12). Despite a strong biologic rationale, this open-label, randomized phase III study did not improve RFS compared with surgery alone (HR = 0.94; 95% CI, 0.74-1.21; *P* = 0.32), highlighting the challenges associated with integrating neoadjuvant and adjuvant immune checkpoint inhibition in resectable RCC (12). One potential explanation for the negative result is the trial design, in which only a single neoadjuvant nivolumab dose was administered before surgery, which may have been insufficient to induce robust immune priming prior to tumor resection. The discordance between KEYNOTE-564 and other perioperative ICI trials is best interpreted as a context-dependent trial effect rather than evidence of a drug-specific advantage of pembrolizumab over other PD-1/PD-L1 inhibitors. KEYNOTE-564 (13, 48) enrolled a biologically enriched population with clear-cell histology and included M1 NED patients, who appear to derive particularly substantial benefit, while also delivering a full year of adjuvant PD-1 blockade. By contrast, IMmotion010 (49) enrolled a broader and more heterogeneous population, CheckMate 914 Part A (50) combined nivolumab with ipilimumab at the cost of greater toxicity and treatment discontinuation, CheckMate 914 Part B (51) showed that nivolumab monotherapy was also insufficient in this setting, and PROSPER (12) tested a perioperative strategy with only a single preoperative nivolumab dose before surgery. Collectively, these findings suggest that adjuvant efficacy in RCC depends on the interaction among disease biology, risk enrichment, treatment intensity, and exposure duration, rather than representing a uniform class effect across ICIs.

Recent conference-reported phase 3 readouts have already begun to refine the adjuvant RCC landscape beyond pembrolizumab monotherapy. In the RAMPART platform trial (54), adjuvant durvalumab plus tremelimumab demonstrated a disease-free survival benefit versus active monitoring after resection of primary RCC, whereas the durvalumab monotherapy comparison remains pending. This risk-adapted design supports the concept that the benefit of adjuvant immunotherapy may vary according to baseline recurrence risk and reinforces a shift from a uniform high-risk approach toward more granular, risk-layered decision-making. In parallel, the phase 3 LITESPARK-022 study (55) showed that pembrolizumab plus belzutifan significantly improved disease-free survival compared with pembrolizumab alone, although overall survival remains immature and the combination was associated with increased toxicity. Taken together, these results suggest that the next phase of adjuvant development in RCC may depend on rational biologic combinations and improved risk enrichment rather than simple extension of single-agent checkpoint blockade. Because some of these data are currently available through conference presentation rather than full peer-reviewed publication, their interpretation should remain provisional pending mature follow-up and complete reporting (as shown in **Table 4**).

**Table 4:** Ongoing and recently reported clinical trials of adjuvant therapy in RCC.

Phase	Clinical trial	Population	Regimen	Primary endpoint	Estimated completion date
II	NCT06005818 (MRD GATE RCC)	Post-nephrectomy ccRCC, increased recurrence risk based on MRD information.	Pembrolizumab	DFS	2028-09
III	NCT03288532 RAMPART*	Resected primary RCC, intermediate/high-risk definitions (e.g., Leibovich-based)	Active monitoring *vs.* durvalumab *vs.* durvalumab + tremelimumab	DFS	2034-12
II	NCT05768464	non-clear renal cell carcinoma with high-risk recurrence factors	Toripalimab + Axitinib	DFS	2027-12-30
III	NCT05239728 LITESPARK-022*	Resected primary RCC, intermediate/high-risk definitions, including M1 NED	Belzutifan + Pembrolizumab *vs.* Placebo + Pembrolizumab	DFS	2029-09-28
II	NCT07047001	Resected primary RCC, intermediate/high-risk definitions	Vorolanib Monotherapy *vs.* Vorolanib + Toripalimab	2-y DFS	2032-06-30
II	NCT06307431	Resected primary RCC, intermediate/high-risk definitions, including M1 NED	V940 (mRNA-4157) + Pembrolizumab *vs.* Placebo + Pembrolizumab	DFS	2032-06-08

RCC, renal cell carcinoma; ccRCC, clear cell renal cell carcinoma; MRD, minimal residual disease; DFS, disease-free survival; NED, no evidence of disease, *Indicates trials with recently reported conference readouts.

Taken together, KEYNOTE-564 establishes adjuvant pembrolizumab as the current benchmark, with demonstrated improvements in both DFS and OS, whereas negative contemporary trials underscore that efficacy is not class wide across ICIs and is likely contingent on appropriate risk enrichment and underlying disease biology. Several factors may contribute to these divergent outcomes, including differences in patient selection criteria, inclusion of non-clear cell histologies, timing of treatment initiation, and duration of therapy. However, despite these encouraging results, several considerations remain. The current median follow-up of approximately 57 months, while sufficient to demonstrate an emerging OS signal, remains relatively short for RCC, where late recurrences can occur. In addition, the M1 NED subgroup analysis was exploratory and involved a limited number of patients. Interpretation of the OS signal also requires caution because post-recurrence therapies, including PD-1/PD-L1–directed treatment received in the control arm after relapse, may influence long-term survival estimates. Finally, the long-term risk–benefit balance of adjuvant immunotherapy requires careful consideration, particularly given the possibility of persistent immune-related toxicities such as endocrine dysfunction requiring lifelong hormone replacement.

### 4.3 Balancing benefit and overtreatment

In spite of its clear efficacy, adjuvant immunotherapy raises important concerns regarding overtreatment of RCC. A proportion of high-risk patients may never experience recurrence even in the absence of adjuvant therapy, yet they are exposed to immune-related adverse events (irAEs), some of which may be chronic or irreversible. At present, patient selection relies primarily on clinicopathologic risk models, such as Leibovich-based stratification, which are prognostically informative but insufficiently precise to individualize expected benefit from adjuvant immunotherapy (56). Notably, patients with M1 NED appear to derive particularly pronounced benefit from adjuvant pembrolizumab, whereas candidate predictive biomarkers, including PD-L1 expression, have demonstrated limited clinical utility in the adjuvant setting (13, 48). These observations underscore the urgent need for improved risk stratification and biomarker-driven approaches to refine patient selection and minimize unnecessary treatment exposure. In clinical practice, decisions regarding adjuvant immunotherapy for RCC must balance recurrence risk against potential toxicity, patient comorbidities, renal function, and individual preferences, highlighting the importance of shared decision-making, particularly given the prolonged treatment duration and the potential impact of irAEs on quality of life. This is particularly important because current guideline recommendations explicitly emphasize discussion of contradictory adjuvant ICI trial results, the possibility of overtreatment, and the risk of long-term immune-related toxicity when counseling patients about postoperative pembrolizumab. The contrasting outcomes between KEYNOTE-564 and other contemporary trials further highlight that adjuvant immunotherapy efficacy in RCC is not a class-wide phenomenon. Differences in trial design, including patient risk enrichment, histologic composition, treatment timing, and duration of therapy, may critically influence outcomes. For example, KEYNOTE-564 focused predominantly on clear-cell RCC and included patients with particularly high recurrence risk (13, 48), whereas other studies enrolled broader or biologically heterogeneous populations. These discrepancies suggest that the effectiveness of adjuvant immune checkpoint inhibition may depend on both tumor biology and careful clinical trial design, rather than representing a universal therapeutic principle.

When considered collectively, the historical trajectory of adjuvant therapy trials in RCC reveals several conceptual lessons that extend beyond the outcomes of individual studies. First, the repeated failures of cytokine-based and VEGF-targeted therapies underscore a critical limitation of early adjuvant strategies: the assumption that therapeutic activity observed in metastatic disease could be directly translated into the minimal residual disease setting. In reality, the biological context of micrometastatic disease differs substantially from that of established tumors, and agents that primarily exert cytostatic effects may be insufficient to eradicate dormant tumor cell populations. Second, the heterogeneity of outcomes observed across contemporary immunotherapy trials highlights the central importance of patient selection. The success of KEYNOTE-564 contrasts with the negative results of several other immune checkpoint inhibitor studies, suggesting that adjuvant efficacy is not a universal class effect but rather a context-dependent phenomenon influenced by tumor biology, risk enrichment, and trial design.

Taken together, these observations suggest that the central challenge of adjuvant therapy in RCC is not merely identifying active systemic agents, but rather aligning therapeutic mechanisms with the biological characteristics of postoperative residual disease. This perspective supports a shift toward biomarker-informed and risk-adapted perioperative treatment strategies.

## 5 Future directions in precision adjuvant therapy for RCC

### 5.1 Biomarker-driven patient selection

Despite the clinical success of adjuvant pembrolizumab, the central unresolved question is no longer whether postoperative immunotherapy can work in RCC, but rather which patients actually require it. Current selection still relies predominantly on clinicopathologic risk models, which are prognostically useful but biologically imprecise and therefore insufficient to minimize overtreatment. A more robust framework will likely require integration of molecular residual disease assessment, tumor immune contexture, and conventional pathologic risk factors rather than reliance on any single biomarker.

Among emerging biomarkers, ctDNA is one of the most clinically actionable candidates because it directly addresses the problem of occult residual disease. ctDNA has emerged as a particularly attractive biomarker for the detection of minimal residual disease (MRD), enabling the identification of molecular relapse prior to radiographic progression. Across multiple solid tumors, MRD-guided strategies have shown potential to individualize adjuvant treatment decisions and enhance therapeutic precision. Early studies in RCC indicate that postoperative ctDNA positivity is associated with a markedly increased risk of recurrence (57, 58), supporting its utility as a tool for molecular risk stratification. In this context, ctDNA-guided adjuvant therapy could facilitate a paradigm shift from empirically treating all clinicopathologically high-risk patients to selectively treating those with molecular evidence of residual disease. However, RCC-specific prospective MRD-guided evidence remains limited, and ctDNA should not yet be presented as ready for routine clinical decision-making. In RCC, the field is only beginning to move toward prospective MRD-guided designs, as illustrated by studies such as MRD GATE RCC (NCT06005818), which are evaluating whether adjuvant pembrolizumab can be allocated according to postoperative molecular residual disease status.

Beyond ctDNA, immune-related biomarkers such as gene expression signatures, T cell receptor clonality, and characterization of the tumor microenvironment may provide additional insights into responsiveness to immunotherapy. Transcriptomic analyses have identified immune-inflamed RCC subtypes characterized by IFN-γ signaling and dense CD8^+^ T cell infiltration, features that have been associated with increased responsiveness to immune checkpoint blockade (59, 60). These observations suggest that incorporation of immune contexture into postoperative risk assessment may help identify patients most likely to derive benefit from adjuvant immunotherapy. However, such approaches remain exploratory and require validation in prospective, biomarker-driven clinical trials before they can be integrated into routine clinical practice.

By contrast, PD-L1 has not emerged as a reliable predictive biomarker in adjuvant RCC. Benefit from pembrolizumab in KEYNOTE-564 was not confined to a clearly defined PD-L1-positive population. The IMmotion010 trial likewise failed to identify a meaningful interaction between PD-L1 status and benefit from adjuvant atezolizumab, underscoring the inadequacy of single-marker approaches (49). In contrast, exploratory analyses from the IMmotion010 trial suggest that patients with elevated kidney injury molecule-1 (KIM-1) levels may experience greater DFS benefit from adjuvant immunotherapy, particularly when accompanied by immune effector-related transcriptional signatures (61). Collectively, these findings highlight the need for more sophisticated biomarker strategies that integrate tumor genomics, immune profiling, circulating biomarkers, and host-related factors to enable truly personalized decision making for adjuvant immunotherapy in RCC.

### 5.2 Challenges facing treatment optimization

Key unanswered questions remain regarding the optimal implementation of adjuvant immunotherapy in RCC, including the appropriate duration of treatment, the potential role of neoadjuvant immunotherapy, and strategies for managing disease recurrence following prior immune checkpoint inhibition. Although adjuvant pembrolizumab has established a new standard of care for patients with high-risk resected RCC, several aspects of perioperative immunotherapy require further refinement through prospective clinical investigation.

First, the optimal duration of adjuvant therapy remains uncertain. Treatment duration varied substantially across trials and may represent an underappreciated determinant of efficacy. While KEYNOTE-564 (13, 48) and IMmotion010 (49) employed approximately 1 year of continuous therapy, CheckMate 914 (50, 51) used a shorter ~6-month regimen, and PROSPER (12) incorporated minimal neoadjuvant exposure. These differences raise the possibility that insufficient immune priming or inadequate treatment duration may have contributed to negative outcomes in certain trials. In KEYNOTE-564 (13, 48), pembrolizumab was administered for one year, a treatment length selected empirically rather than on the basis of biological rationale. It is therefore conceivable that shorter or risk-adapted treatment durations could preserve clinical benefit while reducing cumulative toxicity, improving tolerability, and mitigating economic burden. Future studies evaluating de-escalation strategies, biomarker-guided stopping rules, or individualized durations based on minimal residual disease status are warranted.

The role of neoadjuvant immunotherapy is also under active investigation. Administering immune checkpoint blockade prior to nephrectomy may enhance antigen presentation, increase tumor-specific T-cell priming, and generate systemic immune memory capable of eradicating micrometastatic disease. Nevertheless, randomized evidence to date has not yet demonstrated clear superiority of neoadjuvant or perioperative approaches over adjuvant-only strategies, underscoring the need for improved patient selection and a more refined mechanistic understanding of perioperative immune modulation (12).

Another emerging clinical challenge involves the management of patients who relapse following prior adjuvant immunotherapy. With the increasing adoption of postoperative pembrolizumab, clinicians are more frequently encountering recurrences after prior PD-1 exposure. Real-world data suggest heterogeneous relapse patterns and limited evidence guiding subsequent therapy selection (62). Whether ICIs can be safely and effectively rechallenged, or whether alternative strategies such as VEGF-TKI combinations, HIF-2α inhibitors, or novel immunotherapeutic targets are required, remains an area of ongoing clinical research (63).

Collectively, these unresolved issues highlight that while adjuvant immunotherapy has meaningfully advanced the management of localized RCC, its future optimization will depend on biomarker-driven personalization, improved perioperative trial design, and evidence-based strategies for post-adjuvant relapse management.

### 5.3 Novel combinations and emerging targets

Future clinical trials are increasingly focused on rational combination strategies that integrate immunotherapy with targeted agents or novel immune checkpoint pathways, with the goal of enhancing antitumor efficacy while minimizing treatment-related toxicity. As adjuvant immune checkpoint inhibition becomes progressively incorporated into the management of high-risk resected RCC, optimization of combination strategies will be essential to improve outcomes beyond current monotherapy standards.

**Figure 2:**
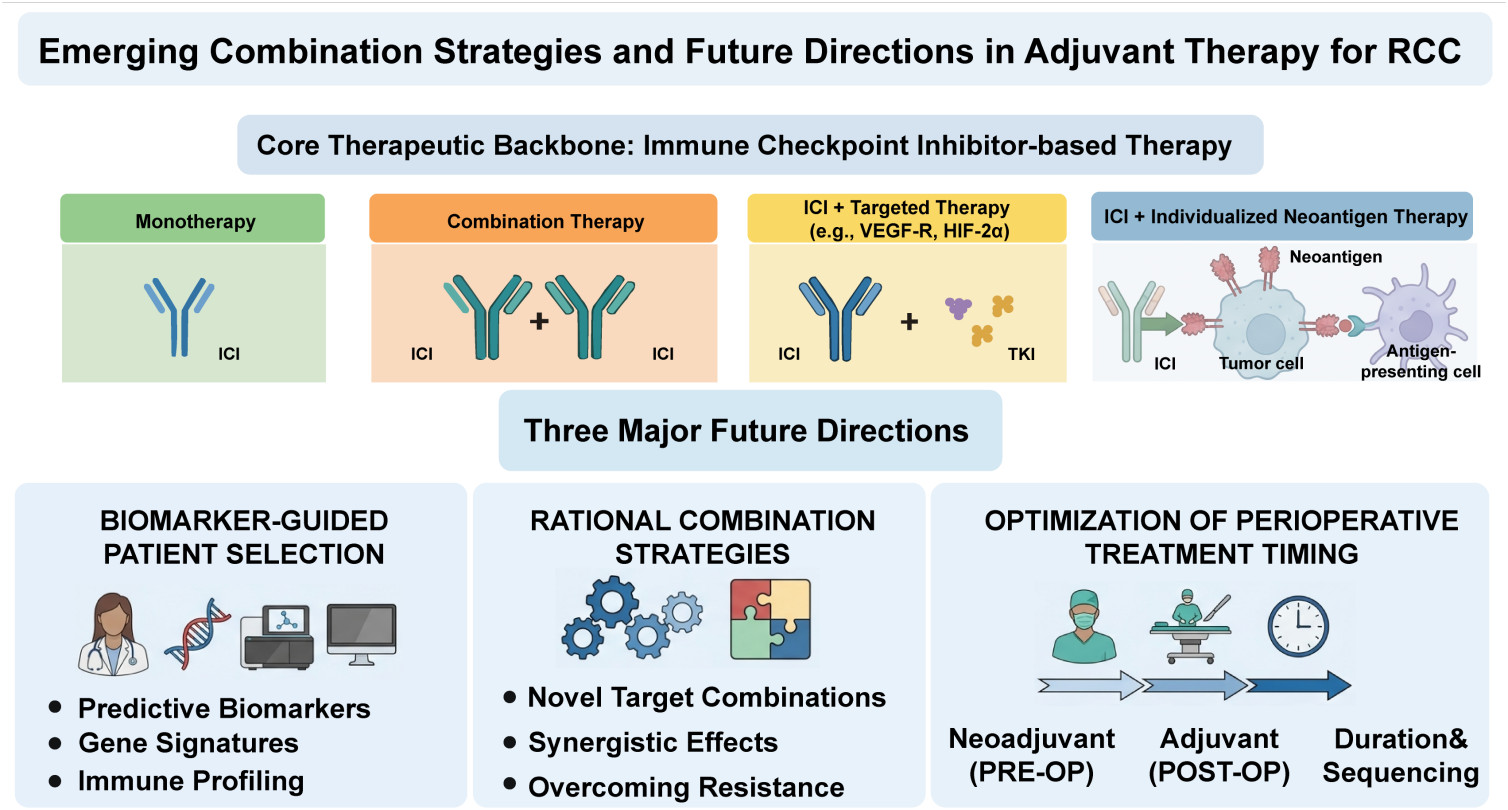
Emerging combination strategies and future directions in adjuvant therapy for RCC. The core therapeutic backbone of adjuvant RCC treatment is ICI-based therapy, which includes monotherapy and various combination approaches such as ICI-ICI combinations, ICI combined with targeted therapies (e.g., VEGFR or HIF-2α inhibitors), and ICI-based individualized neoadjuvant strategies. Future development in this field is focused on three major directions: (1) biomarker-guided patient selection, including predictive biomarkers, gene signatures, and immune profiling; (2) rational combination strategies aimed at enhancing synergistic effects and overcoming therapeutic resistance; and (3) optimization of perioperative treatment timing, including neoadjuvant (preoperative) and adjuvant (postoperative) settings, as well as treatment duration and sequencing. RCC, renal cell carcinoma; ICI, immune checkpoint inhibitor; VEGFR, vascular endothelial growth factor receptor; HIF-2α, hypoxia-inducible factor 2α; TKI, tyrosine kinase inhibitor; PRE-OP, preoperative; POST-OP, postoperative.

To further improve therapeutic benefit, emerging adjuvant approaches are investigating combinations of immune checkpoint inhibitors with targeted therapies, particularly agents inhibiting the vascular endothelial growth factor (VEGF) pathway (as shown in **Figure 2**). This rationale is supported by prior clinical experience with VEGF-targeted therapies in RCC, as summarized in **Table 2**, which highlights both their biological activity and limitations in the adjuvant setting. Preclinical and translational studies suggest that VEGF signaling not only promotes angiogenesis but also contributes to immune evasion (29). Accordingly, VEGF pathway inhibition may enhance the activity of checkpoint blockade and provide a mechanistic basis for combination strategies in the peri-operative setting (64). A review of ongoing and planned studies registered on indicates that future development of adjuvant therapy for RCC continues to be predominantly centered on ICI-based strategies, either as monotherapy or in rational combination regimens. This trend underscores the sustained centrality of immunotherapy in the evolving adjuvant treatment landscape and reflects ongoing efforts to optimize efficacy through biomarker-guided selection and synergistic therapeutic approaches. The ultimate clinical role of these emerging strategies will need to be defined by longer follow-up and full publication of ongoing studies.

In addition, next-generation immune checkpoints such as lymphocyte activation gene-3 (LAG-3), T-cell immunoglobulin and ITIM domain (TIGIT), and T-cell immunoglobulin and mucin-domain containing-3 (TIM-3) are under active clinical development and may provide complementary mechanisms to overcome T-cell exhaustion and adaptive resistance. Comprehensive immune-profiling studies in RCC have demonstrated expression of these inhibitory receptors within tumor-infiltrating lymphocytes, supporting their biological relevance and potential as therapeutic targets (65). However, the incorporation of these agents into the adjuvant setting warrants particular caution. Patients receiving adjuvant therapy are clinically disease-free following nephrectomy and may therefore be especially vulnerable to cumulative immune-related adverse events. As a result, careful evaluation of toxicity, treatment duration, and biomarker-guided patient selection will be essential before such intensified regimens can be widely adopted.

Taken together, these ongoing studies suggest that the next phase of adjuvant therapy development in RCC will likely focus on three interconnected directions: biomarker-guided patient selection, rational combination strategies, and optimization of perioperative treatment timing (as shown in **Figure 2**).

### 5.4 Limitations of this review

This review has several limitations. First, some of the concepts and clinical data discussed here overlap with previously published reviews (66, 67) on RCC and postoperative management, including recent overviews of renal cell carcinoma biology, risk stratification, and adjuvant treatment evidence. Second, the perioperative RCC field is evolving rapidly, and some emerging studies discussed herein are currently available only through conference presentations or early reports, which may limit the stability of interpretation until full peer-reviewed publication. Third, cross-trial comparisons should be interpreted cautiously because differences in eligibility criteria, histologic composition, endpoint definitions, treatment exposure, and follow-up duration may confound direct comparisons. Nonetheless, we believe that a narrative, cross-era synthesis remains useful for identifying recurring design pitfalls, interpreting discordant immunotherapy results, and framing future biomarker-driven strategies.

## 6 Conclusion

In conclusion, the history of adjuvant therapy in RCC provides a clear set of lessons. The repeated failures of cytokine-based and VEGF-targeted strategies illustrate the limitations of empirically extrapolating activity from metastatic disease to the postoperative setting. In contrast, the success of pembrolizumab demonstrates that effective adjuvant therapy requires alignment among tumor biology, therapeutic mechanism, and appropriate patient selection. However, important challenges remain, including overtreatment, long-term immune-related toxicity, limited biomarker guidance, and uncertainty regarding optimal management after prior adjuvant ICI exposure. Future progress in RCC will therefore depend less on simply expanding treatment to all high-risk patients and more on developing biomarker-informed, risk-adapted, and biologically rational perioperative strategies.
